# Supplementary material for: Exploring quadriceps activity variations in women: the role of seated posture during stair climbing
Source: PeerJ. 2024 Nov 22;12:e18547. doi: 10.7717/peerj.18547 (PMC11587870; doi:10.7717/peerj.18547)
Supplement: Supplemental Information 1 [file peerj-12-18547-s001.docx]

IPAQ: 1(low), 2(min), 3(high)

D_leg: 1(Right), 2(left)

Group: 1(SA group), 2(SLC group), 3(SAC group)
